# A Narrative Review on the Role of Acids, Steroids, and Kinase Inhibitors in the Treatment of Keratosis Pilaris

**DOI:** 10.7759/cureus.18917

**Published:** 2021-10-20

**Authors:** Shreya Reddy, Hetal Brahmbhatt

**Affiliations:** 1 Biochemistry, Creighton University, Omaha, USA; 2 Psychiatry, Mercy General Hospital, Sacramento, USA

**Keywords:** kinase inhibitor, steroid, glycolic acid, salicylic acid, keratosis pilaris

## Abstract

Keratosis pilaris is a common and benign genetic skin disorder that results in patches of rough bumps on the skin, with varying degrees of reddening and inflammation. These bumps in the skin are caused by the dead skin cells that plug the hair follicles. Keratosis pilaris often manifests in small, hard bumps on the legs and arms. A common treatment method for many acne conditions, including keratosis pilaris, is acid, which has shown promise in recent years. This review examines the use and success of multiple types of acids, steroids, and kinase inhibitors in clinical and non-clinical settings to treat and understand keratosis pilaris. In the treatment of keratosis pilaris, acid primarily works by breaking down the dead skin cells that clog the hair follicles. Some types of steroids have anti-inflammatory properties that have proven useful in minimizing the appearance of acne. Kinase inhibitors control important skin cell functions, such as cell signaling, metabolism, division, and survival, which undoubtedly affect the appearance of skin as a whole. The known impact of acid, steroids, and kinase inhibitors on keratosis pilaris is underestimated and should be given more attention by healthcare industry leaders.

## Introduction and background

Keratosis pilaris is a common, life-long skin condition that affects approximately 40% of adults and 50-80% of adolescents. This condition is more widespread in females than in males [1]. Keratosis pilaris often becomes more exaggerated at puberty but tends to become less pronounced with increasing age. Seasonal variation is sometimes noted, as in the winter, dry skin becomes more prevalent and can worsen the condition for some patients. In some cases, the disorder can be cosmetically deforming and psychologically discomforting.

Keratosis pilaris appears as rough folliculocentric keratotic papules that typically occur on the upper arms and thighs; however, other locations have also been reported. It is a genetic disorder of keratinization of the hair follicles, with approximately 30-50% of patients having a family history [1]. The disease is most often caused by a buildup of keratin, and in the majority of patients, the process of keratinization is believed to be flawed. An excess of skin cells builds up close to individual hair follicles, and the individual papules are a result of the hair being trapped under the excess skin cells and debris, as they are not able to reach the surface [2]. Frequently, coiled hair may be visualized under the papule.

A well-known treatment for keratosis pilaris is the use of laser therapy. During laser treatment sessions, the skin is subjected to bright lights. These bright lights penetrate the skin and selectively target the melanin in the hair follicle [3]. The melanin absorbs the energy and destroys the hair follicle [3]. However, laser treatments are often expensive and are not easy for the average person to access, in addition to further problems that could result such as swelling and blisters [3]. This narrative review discusses other methods that have been used to treat keratosis pilaris that could prove to be more beneficial.

This paper aims to explore the effects of acids, steroids, and kinase inhibitors on keratosis pilaris. A literature search of articles published between 1955 and 2021 using PubMed was performed using the keywords “keratosis pilaris,” “acid,” “kinase inhibitor,” and “steroid.” Articles that included infants, the elderly, allergies related to acids and steroids, existing history of skin surgeries, and skin trauma were excluded. Titles and abstracts were analyzed to ensure patients were not in a compromised state before the treatment of keratosis pilaris. This review included case studies and group studies. The articles were analyzed for the type of treatment used for keratosis pilaris, the dosage or concentration of the treatment method, and the overall results on keratosis pilaris. Results from the studies were analyzed in a mixed-methodology manner using both quantitative and qualitative analysis to create a holistic review. The quantitative analysis included percentages of effectiveness in some of the treatments, such as how many patients noted improvements in their condition. The qualitative analysis included descriptions of visual elements of keratosis pilaris, such as the examination of the appearance of the keratotic papules. A total of 49 articles were identified, of which 33 were finally reviewed. Only articles that were available online and in English were included in this review.

## Review

Acid

Acid is a popular component of several acne-fighting products as it can help unclog the pores. For instance, glycolic acid works to break the bonds between the outermost layer of the skin, which consists of dead skin cells, and the dermis. This results in a peeling effect which leads to fewer dead skin cells and oil in the pores, resulting in clearer skin [4]. Glycolic acid also eliminates excess accumulation of keratinocytes, accelerating melanin metabolism, promoting the shedding and renewal of epidermal cells, stimulating collagen production, and making the skin brighter [5]. Moreover, glycolic acid is also a very common alpha-hydroxy acid, which works to loosen old and dead skin cells, revealing new skin cells. Scarring is also shown to improve with the use of glycolic acid, setting it up as a good method for treating keratosis pilaris [6]. Salicylic acid is also a well-known ingredient in skin-clearing products and targets keratosis pilaris and other acne disorders by limiting cohesion between keratinocytes [7]. Salicylic acid has been shown to produce the benefits of skin brightening without the risk of extensively damaging pores. It fights acne by dissolving the dead skin cells that are responsible for clogging pores, improving the appearance of the skin. Because salicylic acid is an oil-soluble beta-hydroxy acid, it can penetrate the lipid layers of the skin [6]. Azelaic acid is a dicarboxylic acid that has also demonstrated effectiveness in combating acne. Azelaic acid, most known for its anti-inflammatory and antioxidant properties, works either in monotherapy or combination therapy to treat post-inflammatory hyperpigmentation [8]. Lactic acid can also be used to treat papules. It has the ability to decompose dead skin cells, opening the clogged pores and giving way for other products, such as salicylic acid, to deeply penetrate the skin. Another acid that can be considered helpful in managing keratosis pilaris and other acne is retinoic acid, which improves the appearance of post-inflammatory hyperpigmentation and reduces atrophic acne scarring [9].

Kinase inhibitors

Kinase inhibitors have also been shown to affect keratosis pilaris and its close variants as they block enzymes known as tyrosine kinases. Because tyrosine kinases help to send growth signals in cells, blocking them stops cell growth and division. Nilotinib is a kinase inhibitor that is responsible for many side effects of alopecia, primarily scarring of the skin. [10]. An additional kinase inhibitor of interest is vemurafenib, which is a BRAF inhibitor that is used to treat metastatic melanoma, a skin cancer in which the pigment-producing cells of the skin become cancerous [11].

Steroids

Steroids are among some of the compounds that affect keratosis pilaris. Topical and systemic steroids help by decreasing inflammation, working directly against acne disorders [12]. According to the studies incorporated in this review, specific examples of steroids that influence acne conditions include triamcinolone, hydrocortisone, mometasone furoate, testosterone, prednisone, methylprednisolone, and clobetasol propionate.

Genetic acids

As previously stated, keratosis pilaris is a genetic condition; therefore, some of the genes producing amino acids can have some impact on keratosis pilaris. For example, fibrinogen alpha chain and guanine nucleotide exchange factor 1 genes code for amino acids that create proteins that have an effect on acne and hyperpigmentation [13,14]. However, this review generally focuses on steroids, kinase inhibitors, and types of acids that the majority of keratosis pilaris and other acne treatments consist of.

Importance of concentration and dosage

While acids, steroids, and kinase inhibitors affect keratosis pilaris, the types (as previously discussed) vary, along with their concentrations and dosages. A concentration that is too low would not necessarily bring about the benefits that acid is supposed to create for the skin. However, if the concentration of acid is too high, there is an increased possibility of inflammatory skin conditions. This will especially affect patients who have a sensitive skin type. The concentration of acid also depends on the type of acid that is used.

Concentration of acid

For instance, salicylic acid is most commonly used at a concentration of 5-6% in treating acne and keratosis pilaris [6,15,16]. In some cases, salicylic acid is also used at lower concentrations such as 1-3% [17]. This is most likely because salicylic acid is an oil-soluble acid, which usually works as it can deeply penetrate the skin. However, if the salicylic acid concentration is too high, the oil-soluble acid can actually clog pores and damage the skin’s barrier. Glycolic acid is most commonly used at a concentration of 50-70%, significantly higher than the concentration of salicylic acid [5,6,16]. Glycolic acid is a water-soluble acid that can exfoliate the skin notably better than most of the other alpha-hydroxy acids. In the treatment of keratosis pilaris, glycolic acid is used at high concentrations because it accelerates the process of cell turnover, which improves the appearance of dark bumps on the skin. Acetic acid has also been used to treat keratosis pilaris at a concentration of 0.1 M, and trichloroacetic acid has been used at 20%; however, both are not as effective as other methods and are not used widely [18,19]. Trichloroacetic acid is most commonly used as a non-invasive skin peel, peeling away dead skin cells to reveal the newer and smoother skin layers below, also improving scarring and skin discoloration, making it a candidate to potentially help keratosis pilaris patients [20]. Azelaic acid and lactic acid have been used at concentrations of 20% and 10%, respectively [7,8,15]. Azelaic acid concentration is usually in the range of 15-20% because it reduces the growth of pore-blocking keratin cells and kills the bacteria associated with acne [21]. Lactic acid is used at a concentration of 10% to promote mild exfoliation, which brightens, smoothens, and evens skin tone, making it ideal for keratosis pilaris patients.

Dosage of steroid and kinase inhibitors

Dosages of steroids are important to discuss for medications as too much or too little can produce unwanted side effects. For instance, testosterone is administered at 1.1-8 nmol [22]. According to the studies included in this review, hydrocortisone is administered at 2.5%, triamcinolone at 0.1%, mometasone furoate at 0.1%, and clobetasol propionate at 0.05% [23,24]. Prednisone and methylprednisolone are administered at 20 mg per day and 6 mg per day, respectively [25,26]. Kinase inhibitor dosages are also important for the same reason. According to the studies included in this review, nilotinib is administered to patients at dosages of either 600 mg or 800 mg [10,27]. The concentration of acids, as well as dosages of steroids and kinase inhibitors, are important for those who suffer from skin issues because the effectiveness of skincare products is linked to the concentration of acid of the skincare regimen and how it affects different skin types.

Results of treatment with acid

Acid has been used for treating and understanding keratosis pilaris for many years. This review encompasses studies of different types of acids used to help keratosis pilaris patients and their individual effects. Azelaic acid is one of the acids that has been experimented with. Patients respond to treatment with azelaic acid, as in one study in this review, 92% of participants showed significant improvement in hyperkeratosis erythema after three months of use [8]. Improvement of hyperkeratosis erythema reduces the appearance of the dark papules, improving the appearance of keratosis pilaris. In another study, azelaic acid improved hyperkeratosis and roughness of the skin by 92% [15]. Glycolic acid has the potential to be helpful in improving keratosis pilaris. In another study, all patients using glycolic acid showed short-term improvement of 8-60% in the overall appearance of the impacted areas of their skin; however, after five years, the therapeutic effect of using glycolic acid was minimal for the majority of patients [15,16]. A serum consisting of 0.5% salicylic acid and 50% glycolic acid showed potential as 90% of patients had overall improvement with bumps and 70-80% of patients had more smooth skin [6]. Salicylic acid is also effective at mitigating the effects of keratosis pilaris. In one study included in this review, salicylic acid improved pigmentation, roughness, and overall appearance by 52% [7,15]. In another study, it was noted to show variable effects; therefore, it is important to recognize that it is not a method of treatment that is always been beneficial for keratosis pilaris patients [28]. Lactic acid appears to be consistently advantageous in diminishing the effects of keratosis pilaris. According to the studies included in this review, lactic acid improved roughness, pigmentation, and overall appearance by 66% [7,15]. Retinoic acid, however, was shown to not be effective for keratosis pilaris patients. In this review, a study involving retinoic acid concluded that it causes an increase in cellular retinoic acid-binding proteins, which is correlated with an increased presence of keratosis pilaris [9]. However, tazarotene, which belongs to a class of retinoids, has shown potential. Upon use of 0.01% formulation of tazarotene, some patients showed gradual fading of the dark bumps over four to eight weeks of treatment [29].

Results of treatment with steroids

Steroids have also shown encouraging results when treating acne. The use of hydrocortisone proved to diminish erythema [23]. Hydrocortisone, coupled with triamcinolone, mometasone furoate, and clobetasol propionate, had a positive effect in a study as lesions showed significant improvement when patients adhered to avoiding heat [24]. Prednisone caused gradual disappearance of lichenoid papules, improving the appearance of keratosis pilaris [25]. However, some corticosteroids actually cause more harm to the patients. In one study, methylprednisolone produced a large number of yellow papules on the face and hyperproliferation of sebaceous glands [26]. The proliferation of the sebaceous glands indicated that it would not be the best treatment for keratosis pilaris as it would likely result in an increased prevalence of hair follicles. In another clinical study, it was concluded that administering testosterone correlates with increased severity of keratosis pilaris [22].

Results of treatment with kinase inhibitors

Kinase inhibitors have also proven to be influential in keratosis pilaris. Vemurafenib helped 48% of patients with metastatic melanoma and keratosis pilaris, while the remaining 52% of patients developed rashes (not due to patient allergies) [11]. In all of the studies incorporated in this review involving nilotinib, it was determined that nilotinib induced increased severity of keratosis pilaris, alopecia, and localized lesions [10,27,30-32]. In addition, genetics plays a role in the components of keratosis pilaris.

Results of treatment with genetic acids

One study concluded that fibrinogen alpha chain genes impact keratosis pilaris because it codes for amino acids that produce the profilaggrin protein, which contributes to keratin and acid levels of the skin [13,33]. Another gene with the potential to influence keratosis pilaris, as shown by a study, is the guanine nucleotide exchange factor 1 gene. Guanine nucleotide exchange factor 1 mutated genes have been found to code for amino acids that possibly correlate to the distinct phenotype of keratosis pilaris [14].

Table 1 presents a summary of previous research on the utilization of acids, steroids, and kinase inhibitors to understand and treat keratosis pilaris.

**Table 1:** Summary of previous research on utilization of acids, steroids, and kinase inhibitors to understand and treat keratosis pilaris. N/A: not applicable; ATP: adenosine triphosphate; M: molarity

Author (year), sample size	Type of acid/steroid/kinase inhibitor	Acid concentration/steroid/kinase inhibitor dosage	Results
Tian et al. (2021) [5], 25	Glycolic acid	50/70%	8-60% improvement (short-term). On 5-year follow-up, no change
Wiegmann and Haddad (2020) [6], 66	Glycolic acid, salicylic acid	50% glycolic acid, 0.5% salicylic acid	90% had overall improvement with acne, and 70-80% had more smooth skin
Kootiratrakarn et al. (2015) [7], NA	Salicylic acid, lactic acid	5% salicylic acid , 10% lactic acid	Salicylic acid improved appearance by 52%. Lactic acid improved appearance by 66%
Searle et al. (2020) [8], 38	Azelaic acid	20%	92% showed significant improvement in hyperkeratosis and erythema
Siegenthaler et al. (1986) [9], 13	Retinoic acid	0.75 mg/kg of body weight per day	Increase in cellular retinoic acid-binding proteins leads to increased presence of keratosis pilaris
Tawil et al. (2017) [10], 1	Nilotinib (kinase inhibitor)	600 mg per day	Nilotinib induced keratosis pilaris
Boyd et al. (2012) [11], 132	Vemurafenib (kinase inhibitor)	N/A	52% of patients treated with vemurafenib had “rashes.” In rest of the patients, it helped with metastatic melanoma and keratosis pilaris
Tay et al. (2002) [12], 12,323	Topical and systemic (steroids)	Topical steroid: 13% Systemic steroid: 1–7%	N/A
Liu et al. (2018) [13], N/A	Amino acid (expression of ATP-binding cassette subfamily A member 12 and fibrinogen alpha chain genes)	N/A	Fibrinogen alpha chain gene coding for profilaggrin protein can impact keratin and acid levels. ATP-binding cassette subfamily A member 12 gene does not have an affect
Zenker et al. (2007) [14], 165	Amino acid (expression of guanine nucleotide exchange factor 1 gene)	N/A	Guanine nucleotide exchange factor 1 mutated gene contributes to distinct phenotype of keratosis pilaris
Maghfour et al. (2020) [15], N/A	Azelaic acid, salicylic acid, lactic acid	20% azelaic acid, 5% salicylic acid, 10% lactic acid	Azelaic acid improved hyperkeratosis and roughness by 92%. Salicylic acid improved pigmentation and roughness by 52%. Lactic acid improved pigmentation and roughness by 66%
Pennycook et al. (2021) [16], N/A	Salicylic acid, glycolic acid	6% salicylic acid, 70% glycolic acid	Glycolic acid improved the appearance of keratosis pilaris
Alai (2020) [17], N/A	Salicylic acid, kojic acid, azelaic acid, aminolevulinic acid, lactic acid, and glycolic acid	2–3% salicylic acid, 15–20% azelaic acid, 10–20% glycolic acid, rest N/A	N/A
Dekio et al. (1989) [18], 1	Acetic acid	0.1 M	Produced fibrous proteins that contribute to keratosis pilaris
Saadi (2021) [19], 20	Trichloroacetic acid	20%	N/A
Barth et al. (1988) [22], 227	Testosterone (steroid)	1.1–8.8 nmol	Higher testosterone correlated to increased severity of keratosis pilaris
Edelstein (1955) [23], 1	Hydrocortisone (steroid)	2.5%	Diminishing of erythema
Jeffries et al. (2009) [24], 1	Triamcinolone, hydrocortisone, mometasone furoate, clobetasol propionate (steroid)	Triamcinolone: 0.1%, Hydrocortisone: 2.5%, Mometasone furoate: 0.1%, Clobetasol propionate: 0.05%	With heat avoidance, lesions showed significant improvement
Zegarska et al. (2010) [25], 1	Prednisone (steroid)	20 mg per day	Gradual disappearance of lichenoid papules on extremities
Caytemel et al. (2020) [26], 1	Methylprednisolone (steroid)	6 mg per day	Large numbers of yellow papules on face and hyperproliferation of sebaceous glands
Leong and Aw (2016) [27], 1	Nilotinib (kinase inhibitor)	800 mg per day	Nilotinib induced keratosis pilaris
Shimizu et al. (2016) [28], 1	Salicylic acid	N/A	Variable effectiveness
Gerbig (2002) [29], 20	Tazarotene (acid)	0.01%	Gradually faded and resolved within 4–8 weeks
Oro-Ayude et al. (2020) [30], 1	Nilotinib (kinase inhibitor)	N/A	Nilotinib induced keratosis pilaris
Frioui et al. (2020) [31], 1	Nilotinib (kinase inhibitor)	N/A	Nilotinib induced alopecia and hyperkeratosis
Kowe et al. (2021) [32], 1	Nilotinib (kinase inhibitor)	600 mg per day	Nilotinib induced localized keratosis pilaris lesions
Wang et al. (2018) [33], 14	Amino acid (expression of fibrinogen alpha chain gene)	N/A	Created profilaggrin which contributes to keratosis pilaris due to influence on keratin

## Conclusions

According to studies included in this review, it is clear that acid demonstrates both promise and potential for further treatment and knowledge of keratosis pilaris, in addition to the limited potential of steroids and kinase inhibitors. The ability of different types of acids to primarily exfoliate the skin and penetrate deep into the pores, or allow other skincare products to deeply penetrate into the pores, coupled with specific steroids and kinase inhibitors that can reduce the severity of the condition seems to be the root of prospective treatments of keratosis pilaris. Further studies should be conducted with different types of acids, steroids, and kinase inhibitors to understand their long-term effects on mitigating the dark bumps of keratosis pilaris. These studies should be given more priority by dermatologists and healthcare leaders as a viable and practical treatment for keratosis pilaris, as well as other disorders closely related to keratosis pilaris.
